# Evaluation of the educational technology "Caring for dependent people" by family caregivers in changes and transfers of patients and tube feeding**^1^**

**DOI:** 10.1590/1518-8345.0846.2774

**Published:** 2016-08-18

**Authors:** Maria José Lumini Landeiro, Heloísa Helena Ciqueto Peres, Teresa Vieira Martins

**Affiliations:** 2 PhD, Adjunct Professor, Escola Superior de Enfermagem, UNIESEP, Porto, PT, Portugal.; 3 PhD, Full Professor, Escola de Enfermagem, Universidade de São Paulo, São Paulo, SP, Brazil.; 4 PhD, Professor, Escola Superior de Enfermagem, UNIESEP, Porto, PT, Portugal.

**Keywords:** Caregivers, Nursing, Educational Technology

## Abstract

**Objective:**

to assess the contributions of interactive educational technology "Caring for Dependent People" in the development of knowledge to family caregivers of dependent people in a household context and their satisfaction in its use.

**Method:**

quasi-experimental study, not randomized, of the before and after type, with a convenience sample of 65 family caregivers, from two Medicine services of a hospital in Porto, Portugal. The Control Group consisted of 33 family caregivers and the Experimental Group of 32, identified by consecutive sampling. The experimental group had access to educational technology at home. Data were collected by socio-demographic, satisfaction and evaluation of knowledge questionnaire, about how to feed by nasogastric tube, positioning and transferring the dependent person. The assessment in both groups had two moments: initial, during hospitalization and one month after discharge.

**Results:**

the experimental group had a larger increase in knowledge related to the use of the educational technology. In the control group the knowledge did not differ in the two evaluation time points.

**Conclusion:**

these results confirm the improvement of interactive educational technologies and in the training of family caregivers to care for dependents. This technology successfully met the technical quality and learning needs of caregivers, and was considered easy and stimulating.

## Introduction 

One of the challenges for health in Europe is to find solutions to the aspects emerging from the aging of population. The Strategy Europe 2020 presents social challenges particularly in health and demographic change and wellbeing. A key response to this rapid change in the age structure consists in promoting the creation of an active aging culture throughout life, thus ensuring that the people close to sixty, or more, have opportunities for employment and active participation in social life and family^1^. The World Health Organization defines active aging as the process of optimizing opportunities for health, participation in society and security in order to improve the quality of life as people age^1^. Europe is known for its capacity for innovation and its future rests in the creation of a rich initiative in which stands out the "Innovation Union" considering innovation^2^ as "*the ability of individuals, companies and whole nations create, continuously, the future they want*", being part of this strategy the production of smart, sustainable and inclusive growth that may translate at the tertiary level in the quality of care for the elderly by social innovators^2^.

In the context of Europe 2020, the European Commission proposed the creation of an Innovation Partnership for Active and Healthy Aging^3^ (European Innovation Partnership on Active and Healthy Ageing - EIP-AHA) created under the flagship initiative "Innovation Union". According to the proposal for decision (COM (2013) 0500- C7-0219 / 2013 - 2013/0233 (COD)^4^, in this EIP-AHA Partnership framework the innovative solutions based on information and communication technologies (ICT) should play an important role in achieving the goals by 2020, increasing by two the number of years of healthy life and improve the citizens' quality of life and efficiency of health systems in the Union. Its strategic plan execution set priorities to accelerate and enhance innovation in the field of active and healthy aging throughout the Union in three domains: disease prevention and health promotion, health care and treatment, independent living and social inclusion^3^ This project has the vision to contribute, through educational technology, to the development of knowledge and skills of family caregivers.

In this way, it is aligned with the EIP-AHA^3^ and part of the C2 group - *Development of interoperable independent living solutions of European Innovation Partnership on Active and Healthy Ageing* of the European Commission, pledging to contribute with their results to achieve the deliverables of this group, described in the *Action group-Specific Form - Invitation for Commitments* ^5^. This commitment has to do in this phase of the project, with the "*Development of toolkit / guidance for user empowerment*" which consists in the production and implementation of tools/guidance for users' training, incorporating co-creation, raising awareness and building reliable and friendly tools.

In a research study^6^ conducted, it was found that there is no strong evidence of an increased training of family caregivers and dependents when subjected to interventions through the use of educational technologies than those who are subjected to another type of intervention. However, there are clear improvements in various outcome measures that indicate a benefit when implementing education programs over the internet.

This project aims to bridge the gap between the advances of technological research and the practical needs of family caregivers, through the development of communication solutions and easy access to information via internet. The interactive educational technology "Caring for Dependent People" is a platform that aims to assist family caregivers in the self-care environment of a dependent person with greater security and autonomy. This technology covers the following topics: self-care, feeding via a nasogastric tube, positioning and transferring. The tool contains six different menus: intro, themes, photo gallery, contacts, useful links and tool map. The multimedia (image, video, audio and text) are present in the six menus. The family caregiver opens the tool from an internet browser (explorer or chrome).

## Objective 

To evaluate the contributions of the educational interactive technology: "Caring for Dependent People" in the development of knowledge in the family caregivers at home, and evaluate their satisfaction with its use.

## Method

Quasi-experimental, not randomized study, of the before and after type. The sample of convenience consisted of 65 family caregivers of patients admitted with functional dependence assessed by the Barthel index, identified over a period of five months in two Medicine Services of a hospital in the Porto region, Portugal. Inclusion criteria were to accept to participate in the study, being aged 18 years or more, to have access to the internet at home with basic skills to handle information technology or support of a family member or significant other meeting these conditions and having under their responsibility one dependent person with recent hospitalization.

The experimental group (EG) began with 38 family caregivers. However, there were five losses by the death of the dependent person and a loss by disease of the family caregiver. In the control group (CG), 36 family caregivers were included but for the death of two dependent people and the institutionalization of another, it remained with 33 family caregivers. 

To prevent the participants of the CG to had contact with those of the EG, it was decided to carry out the selection of participants in both groups in different services and during the first 70 days of the start of data collection. After this period the groups were reversed in the middle of the selection period and the service where the EG was identified was then identified as the CG and vice versa. Only the EG had access to a link http://online.esenf.pt/cuidarpessoadependente/ that allowed access to the interactive educational technology at home. Both groups underwent two stages of evaluation with a set of tools that allowed assessing the knowledge. The family members of EG completed a satisfaction questionnaire on the use of educational technology.

The instruments used for data collection were:

- Sociodemographic questionnaire for family caregivers and dependent person built specifically for this study with relevant variables for the characterization of the participants: age, gender, occupation, marital status, years of education, number of children, cohabitation and clinical variables reason for hospitalization, reason of dependence and previous hospitalizations.

- The Barthel Index (BI), adapted Portuguese version^7^ consisting in ten questions concerning the independence in performing activities of daily living, with the lowest score related to greater dependence. The cutoffs adopted are those proposed by the authors: <8 points (totally dependent), 9 to 12 points (severe dependence), 13-19 points (moderate dependence) and 20 independent^7^.

- The *questionnaire for evaluation of the knowledge* of family caregivers was built specifically for this study. It included ten questions to assess each segment (nasogastric tube, positioning and transfer). The questions were prepared taking into account the main care points in each of these procedures. The internal consistency of the instrument was assessed by *Cronbach's alpha*. The assessment questionnaire of knowledge on how to "transfer patients" presented an alpha value of 0.79 in the first and 0.83 in the second evaluation. The questionnaire assessing knowledge about the care in "positioning the patient" showed an alpha coefficient of 0.72 and 0.65 in the first and second evaluation respectively. These figures are indicative of reasonable to good internal consistency^8^.

- The *questionnaire of satisfaction of family caregivers* was adapted from the instrument "Questionnaire for User Interaction Satisfaction" (QUIS) reduced version 7.0^9^. The instrument consists of two parts: the family caregiver characterization data that included socio-demographic and technological variables, such as age, sex, education level, marital status, skill level in computer use, frequency of use of new technologies and time spent with educational technology; the second part consisting in the evaluation of satisfaction with the use of educational technology and containing 7 groups with 21 specific questions. Thus, the family caregiver, for each question from each group and through a measurement scale of 9 points (positive adjectives in the right and negative side on the left), assessed the *overall reaction to educational technology*, the *screen*, the *terminology and the information system*, the *learning*, the *capability of the educational technology*, the *navigation guide and online help* and finally the *multimedia group*.

Data collection carried out by the researcher and associates (nurses) of the Medicine services, took place from March to July 2014. The first evaluation of baseline was while patients were still hospitalized and the second evaluation was performed during a home visit, which had been agreed upon and happened one month after the clinical discharge. The evaluation of the contribution of interactive educational technology was performed using the evaluation intra- and inter-subject. Both groups had the same procedure except that the interactive educational technology was not presented to the control group, nor provided a guide for navigation. A telephone contact between the 1st and 2nd contact was done for the EG, in order to identify whether the participants had any problems or doubts with interactive educational technology. After the second evaluation, the CG was granted access to the tool. 

For the data analysis the information was processed through the statistical program SPSS version 22.0 IBM^(r)^ using parametric statistics^8^. The t-Student test was used to compare the averages between independent samples comparing EG with the CG in relation to the evaluation questionnaires knowledge. The intra-subject comparison was performed using the Student t test for paired samples to compare variations within the groups in the two evaluated periods. To assess the accuracy and reliability of each of the knowledge questionnaires, it was used the Cronbach's alpha. This procedure is the most used and reported in the literature, and its value is calculated based on the average of the interrelations among all test items^8^. According to the same author, a good internal consistency exceeds an alpha of 0,80. The Pearson correlation^8^ was used to calculate the strength of association of continuous variables, specifically to analyze the relationship between the variable "Schooling" and the knowledge acquired (correlation coefficient of 1 means a positive perfect correlation between the two variables , greater than 0,7 is strong, 0,4 to 0,7 moderate and under 0,3 weak)^8^.

The nonparametric test of chi-square (χ^2)^ was used to analyze the association between nominal variables by comparing the distribution in two independent groups (EG and CG) for the variable dependency in different self-care of the dependent person and some socio-demographic variables. The analysis of socio-demographic data was performed using descriptive statistics using measures of central tendency and dispersion. When comparing the groups it was considered a significance level of p <0.05.

The study was approved by the Research Coordination Office (DEFI) and by the Hospital of Porto Central Ethics Committee (CHP) under Ref 157/11 (107-DEFI / 137-CES). In this study, the rules of conduct referred to in the Helsinki Declaration and the national legislation were respected, and the confidentiality of the data collected was guaranteed. Caregivers who met the inclusion criteria were selected and invited to participate. All family caregivers who agreed to integrate the study signed a Free and Informed Consent form.

## Results

The study participants were 65 family caregivers, 33 belonging to the CG and 32 to the EG, mostly females: 27 (EG) and 29 (CG). The average age at the EG was 57,69 years and 56,64 years in the CG, with an average of 8,34 years of schooling in the EG and 7,85 years in the CG. Regarding the occupation of the 65 family caregivers, 25 (38,5%) were in retirement or preretirement situation, 23 (35,4%) were active, 16 (24,6%) unemployed and 1 (1,5%) handicapped. Of the total family caregivers, the majority 36 (55,4%) were daughters and 12 (18,5%) spouses. On the whole, 44 (67,7%) were married, 48 (73,8%) lived with the dependent person and were providing assistance for an average of 5,4 years (EG) years and 3,8 years (CG). Moreover, the majority 61 (93,8%) tended for the dependent person and only four (6,2%) were first-time caregivers. In the total sample, 40 (61,5%) of family caregivers said there was no family support to take care of the dependent person and 25 (38,5%) reported that the siblings or children provided this support. 

Regarding the dependent person, the main reasons for hospitalization in the Medicine service were respiratory, cardiac and urinary problems. The largest share, 43 were female (66,2%), 22 (33,8%) were male, with an average age of 80,97 years (EG) and 78,85 (CG). Regarding the degree of dependence, the majority 47 (72,3%) were totally dependent; 14 (21,5%) had severe dependence and only 4 (6,2%) with moderate dependence; with a dependence time of less of one year (24,6%), between one and four years (43,1%) and from four to 27 years (32,3%) with an average of 4,4 years of being dependent. The main causes of dependence were mental and behavioral disorders 31% (Alzheimer's, dementia), nervous system diseases 7,5% (Parkinson) and diseases of the circulatory system, in particular stroke (16,9%), among others. There were no statistical differences in the distribution of age, gender, educational level of family caregivers, time being caregiver and age of the dependent person, between EG and CG. 

In both groups, participants with higher educational level have shown a higher level of knowledge in the care of feeding through a nasogastric tube, in positioning and transferring patients. In relation to nominal variables sex, marital status, relationship to the dependent person, the application of Chi-Square also did not show significant differences between EG (32) and CG (33). Thus, the groups showed to be equivalent at the baseline as presented in Table 1.

In Table 1 are specified the results of the initial evaluation and after the knowledge intervention in the first moment and after intervention

**Table 1:** Evaluation of the initial knowledge and after intervention between control group and experimental group, by average, standard deviation and *t*-test values. Porto. Portugal, 2014

Knowledge	EG*	CG^†^	*t* (p) ^§^
	A (SD)^‡^	A (SD)^‡^	
Positioning before intervention	n=32 5,8 (2,7)	n=27 5,4 (3,1)	0,53(ns)^||^
Positioning after intervention	n=32 8,3 (2,0)	n=27 4,9 (2,8)	5,35(0,0001)
Transferring before intervention	n=32 6,1 (2,3)	n=31 5,6 (2,5)	0,79(ns)^||^
Transferring after intervention	n=32 8,8 (1)	n=31 6,0 (2,1)	6,65(0,000)
Care w/nasogastric tube before	n=5 8,4 (1,3)	n=2 8,5 (0,7)	na^ｶ^
Care w/nasogastric tube post-intervention	n=5 8,6 (1,5)	n=2 8,0 (1,4)	na^ｶ^
Global knowledge before intervention	n=32 11,9 (4,4)	n=25 11,3 (5,4)	0,50(ns)^||^
Global knowledge post-intervention	n=32 17,1 (2,5)	n=25 11,1 (4,7)	5,76(0,000)

* Experimental Group; †Control Group; ‡Average and Standard deviation; § *t-Student* Test*;* ||non significant; ｶnon evaluated

It is noted in Table 1, that while the initial assessment groups are equivalent in knowledge of the different areas evaluated, after the intervention the EG shows to have more knowledge in each area evaluated in the second assessment, something that did not occur with the CG.

In intra-subject analysis (Table 2) it is observed that the EG presents more solid and consistent knowledge after contact with the interactive educational technology in all the assessed areas (knowledge related to transfer, positioning and global knowledge). In the control group, the knowledge got worse in the second moment, with the exception of knowledge related to transfer, showing how the conveyed information on how to act in care, faded away.

**Table 2:** Intra-subject evaluation related to knowledge in the control and experimental groups, mean values, and standard deviation values of the Student's *t* test for paired samples. Port. Portugal, 2014

Knowledge	Before intervention	After intervention	t (p)
	A (SD)	A (SD)	
Positioning	EG* n =32 5.81(2,67)	8,34(1,96)	-9,03(0,0001)
	CG^†^ n= 27 5,41(3,13)	4,89(2,83)	3,02(0,006)
Transfer	EG n =32 6,09(2,25)	8,78 (0,99)	-8,29(0,0001)
	CG n =31 5,61(2,52)	5,97(2,14)	-1,48(0,14)
Global knowledge	EG n=32 11,90(4,44)	17,13(2,48)	-10,09(0,0001)
	CG n=25 11,25(5,42)	11,14(4,71)	0,295(0, 771)

*Experimental Group; †Control Group

Given the low number of participants in completing the questionnaire relative to feeding through a nasogastric tube, it was not possible to analyze the statistical differences.

There was a moderate positive correlation between educational status and total knowledge before the intervention (r = 0,528; p = 0,000) and also with the total knowledge after the intervention (r = 0,407; p = 0,002).

In the EG (32) it was assessed the satisfaction with the use of technology. Regarding the ability to use the computer, 18 (56,3%) of family caregivers reported having almost none or little skill, 8 (25%) fair or good and 6 (18,8%) good skills. On the question of whether they frequently used new technology to search information to health care, 19 (59,4%) said they did not and 13 (40,6%) responded that they were used to do so. The time spent by family caregivers with the use of technology education varied: 18 (56,3%) spent from 1 to 4 hours, 13 (40,6%) spent from 4 to 10 hours and 1 (3,1%) more than ten hours.

There was a positive asymmetric distribution in the values of satisfaction, in the same way that in most of the items (14) the minimum score was 7 and the maximum score of 9. In the multimedia group, the item *duration of the film* had an average of 5,84 with a minimum grade of 5 and a maximum score of 8, meaning that most family caregivers considered as appropriate the duration of the films. The family caregivers identified as a critical point in the tool *failures in technology* (minimum 3 and M = 8,66), followed by the sound quality (minimum score of 4 and M = 8,03). The item *reliable technology* was considered the most appreciated, reaching a minimum score of 8 and a maximum of 9 (M = 8,56).

## Discussion

The results show that most family caregivers of dependent people are women living in the same household and who become caregivers for their parents or husbands. Thus, participants in this study have the same profile described in other studies^10^ ^-^ ^13^ that describe aspects of the of the person that usually takes care of the dependent person: gender (primarily women), kinship (daughters , spouse), physical proximity (live together) and low education. We also had seven husbands, a son and a brother as caregivers. In studies^11^ ^-^ ^12^, the results point to an increase in male caregivers, elderly spouse caregiver and inclusion of relatives/caregivers as sisters/brothers, grandson/granddaughter and nephew/nieces. 

Regarding the profile of dependents, there is a predominance of female elderlies. In this study, the minimum age of dependents was 27 years and a maximum of 97, with an average of 80 years in both groups. These results corroborate studies^11^ ^-^ ^12^ ^,^ ^14^ ^-^ ^15^ in Brazil and in Portugal, showing that the percentage of dependent people increases in the older age groups. Analyzing the situations of dependency it is verified that the transition from independence to dependence condition was due mainly to chronic diseases with implications for the impairment of functional capacity. One recent study^15^ shows similar results when looking to identify the cause of dependency in the elderly. This dependence has an average duration of 4,4 years, similar to other reports^14^ ^-^ ^15^. 

It was also found that the introduction of an interactive educational technology contributed to the development of knowledge in the family caregivers in order to take care of the dependent person. These results are similar to an international multicenter study^16^ conducted with family caregivers of patients with Alzheimer's disease who reported that the use of ICT can be very useful to improve the quality of life, care and safety, monitoring rest and movement, use of medication, environmental conditions and emergency communications. These data confirm that family caregivers considered that the *Smart Home for Elderly People* (HOPE) technology system could be very useful to improve the management of patients.

Other studies^17^ ^-^ ^18^ have also shown that people who received instruction only from written material presented worse performance than people who received learning by using technology. In the same way, patients who received internet-based education improved their level of knowledge more significantly than those who underwent face-to-face guidance. 

The present study has shown that there is decrease of the knowledge relating to the care of "positioning the patient" in the CG between the 1^st^ moment of assessment carried out at the hospital and the 2^nd^ time, a month after discharge. This fact confirms the importance of guidance and continuing education by health professionals to family caregivers. This result also highlights the need to strengthen prioritized and essential information during the recovery process immediately after discharge, emphasizing the key role of nurses in the monitoring and management of this process.

The consequences of inappropriate decisions in the field of information transmitted to family caregivers can have the care of dependent persons are remarked, as already reported in some studies^13^ ^,^ ^15^ ^,^ ^19^.

In this context, interactive educational technology applied to health education emerges as an essential and innovative intervention in ensuring the hospital and home transition, to provide support and ongoing guidance to caregivers. It also highlights the importance of leveraged partnerships with healthcare professionals and their interaction with the health system, improving the quality of life and care for dependent person.

It also denotes the power of education considered as a facilitator in the teaching and learning process related with the information about home care. In this sense, given the socio-demographic characteristics of caregivers, access to technology is an expressive reality. A recent study^16^ that describes family caregivers aged ≥ 50 years and with low education considered the ICT as most useful than family caregivers aged <50 years (p <0,0001) and those with high educational level (p <0, 0001). However, the basic skills to use computers to access the Internet and to seek health information, are clearly higher in those with more years of formal education and strongly correlated with the level of e-literacy in health^20^ ^-^ ^22^.

The data obtained by family caregivers were very acceptable and confirmed the overall satisfaction of caregivers with the use of interactive educational technology. They considered this technology as widely satisfactory, stimulating and easy to use. The assessment of the tool was directly related to the quality of it. However, we find similar results in another study^23^ that also evaluated the satisfaction of website users.

## Conclusion 

From the results obtained in this study, we could demonstrate the effective contribution of interactive educational technologies for the development of knowledge in family caregivers. This technology successfully met the technical quality and learning needs of caregivers and was considered easy and stimulating.

The development of knowledge was considered the indicator to assess the impact of interactive educational technology, having been found statistically significant differences in the outcome variables, both in inter-subject evaluation, and in intra-subject evaluation. The difference in results found in the EG allows us to state that the use of educational technology "Caring for Dependents People" contributed to the development of knowledge in family caregivers in the different domains of self-care. It is suggested the expansion of the research subjects, just as the study sites for its implementation.

 These results emphasize the need to include strategies to integrate interactive educational technologies in the training of family caregivers to care for dependents in organizational contexts. It is a challenge to implement and enhance health policies that include training and the application of educational technology and achieve its mastery in the educational processes of family caregivers.
